# Baicalein combined with azoles against fungi *in vitro*

**DOI:** 10.3389/fmicb.2025.1537229

**Published:** 2025-03-13

**Authors:** Mengmin Liang, Qingwen Hu, Junhao Yu, Heng Zhang, Sijie Liu, Jiangrong Huang, Yi Sun

**Affiliations:** ^1^School of Basic Medicine, Health Science Center, Yangtze University, Jingzhou, China; ^2^Department of Clinical Medicine, Yangtze University, Jingzhou, China; ^3^Department of Dermatology, Jingzhou Hospital Affiliated to Yangtze University, Hubei Provincial Clinical Research Center for Diagnosis and Therapeutics of Pathogenic Fungal Infection, Jingzhou, China; ^4^Endocrinology Department, The Third Clinical College of Yangtze University, Traditional Chinese Medicine of Jingzhou Hospital, Jingzhou, China

**Keywords:** baicalein, posaconazole, itraconazole, synergy, *Aspergillus*, dematiaceous fungi

## Abstract

**Background:**

Invasive fungal infections (IFIs) constitute a significant health challenge, particularly among immunocompromised individuals, characterized by a high prevalence and associated mortality rates. The synergistic administration of Baicalein (BE) with azole antifungal agents could potentially herald a novel therapeutic paradigm.

**Materials and methods:**

54 *Aspergillus* strains and 23 strains of dematiaceous fungi were selected. The standard M38-A2 microbroth dilution method was used to test the minimum inhibitory concentration (MIC) and the fractional inhibitory concentration index (FICI) of fungi when BE combined with itraconazole (ITC), voriconazole (VRC), posaconazole (POS) and Isavuconazole (ISV).

**Results:**

BE shows synergistic effects with POS and ITC, with 89.61% and 25.97% of fungal strains. The BE/POS regimen exerted synergistic effects in 87.04% of *Aspergillus* and an impressive 95.65% of dematiaceous fungi. In comparison, the BE/ITC combination showed significantly lower synergy, affecting 33.33% of *Aspergillus* and a mere 8.70% of dematiaceous strains. Antagonistic interactions were sporadically observed with BE in combination with ITC, VRC, POS and ISV. Within the azole class, the BE/POS pairing stood out for its frequent synergistic activity, in contrast to the absence of such effects when BE was paired with VRC or ISV. Highlighting the potential of BE/POS as a notably effective antifungal strategy.

**Conclusion:**

*In vitro*, BE/POS combination emerged as the most effective antifungal strategy, exhibiting synergistic effects in the majority of *Aspergillus* and dematiaceous fungi strains, whereas BE/ITC showed significantly less synergy, and BE with VRC or ISV displayed no synergistic activity.

## Introduction

Invasive fungal infections (IFIs) are a serious type of infectious disease, with higher incidence and mortality rates particularly among patients with immunosuppression or immunodeficiency (Fang et al., 2023). *Aspergillus* species are the main pathogens most frequently isolated from patients with compromised immune function (Badiee and Hashemizadeh, 2014; Pfaller et al., 2021). *Aspergillus fumigatus* and *Aspergillus flavus* are known to be pathogenic, while *Aspergillus niger* and *Aspergillus terreus* are also capable of causing invasive infections (Balajee, 2009; Borni et al., 2024; Hedayati et al., 2007; Morelli et al., 2021; Wang et al., 2024). Dematiaceous fungi, including *Exophiala dermatitidis* and *Exophiala alcalophila*, can cause a variety of infections in immunocompromised individuals (Kirchhoff et al., 2019; Kondori et al., 2024; Revankar, 2007). Azoles have become the mainstay of treatment and prevention for many systemic mycoses, with common medications including ITC, VRC, POS and ISV (Kaushik and Kest, 2018; Peyton et al., 2015). Because of their high infection and mortality rates in immunocompromised patients, as well as the increasing resistance to azole antifungal agents, studying *Aspergillus* and dematiaceous fungi can help explore their resistance mechanisms and provide a theoretical basis for the development of new combination therapeutic strategies (Amona et al., 2022; Djenontin et al., 2023; Puerta-Alcalde and Garcia-Vidal, 2021). Previous research has demonstrated that synergistic combinations of natural products can augment antifungal potency, which may facilitate the discovery of innovative therapeutic approaches to combat fungal infections (Augostine and Avery, 2022; Yang et al., 2023). A wide range of natural flavonoids have been shown to possess antifungal properties (Jin, 2019). Baicalein (BE) is a flavonoid compound widely found in the Scutellaria genus of plants, featuring hydroxyl groups that contribute to its bioactivity (Zhao et al., 2022). Previous studies have confirmed its anti-inflammatory, antioxidant properties, as well as anti-cancer and tumor cell proliferation inhibition effects, while recent research has highlighted the potential antifungal activity of BE (Gupta et al., 2022; Lai et al., 2024; Li Y. Y. et al., 2022; Song et al., 2021; Tuli et al., 2020; Yan et al., 2018). BE exhibits potent antifungal activity against *Candida* species, with a MIC50 as low as 13 μg/mL, and has been proven to inhibit the growth of *Candida* through multiple mechanisms, such as targeting and inhibiting the function of enolase 1 (Eno1) in *Candida albicans*, upregulating the expression of *CPD2*, and inducing apoptosis by targeting ribosomes in *Candida auris* (Da et al., 2019; Li et al., 2024; Li L. et al., 2022; Lv et al., 2022; Serpa et al., 2012). In contrast, the effects of BE on *Aspergillus* and dark-coloured fungi have been less studied. Notably, BE at a concentration of 0.25 mM has been demonstrated to ameliorate *Aspergillus fumigatus* keratitis in mice (Zhu et al., 2021). BE has been shown to exhibit synergistic effects with other antifungal agents, such as fluconazole (FLU), against *Candida parapsilosis* and *C. albicans* (Janeczko et al., 2022; Li L. et al., 2022). In *Candida* species, the combined use of BE and FLU can reduce the MIC values of both antifungal agents, resulting in a better inhibitory effect against fungi (Serpa et al., 2012). Therefore, we hypothesize that combining BE with other antifungal agents could reduce the effective concentration of BE against these fungi, thereby achieving similarly robust antifungal effects as observed against *Candida* species. This investigation further examines the synergistic antifungal efficacy of BE in conjunction with other azole-class drugs, aiming to enhance azoles treatment efficacy and mitigate the development of resistance.

## Materials and methods

### Selection and identification of fungal strains

This study used 54 *Aspergillus* strains [31 strains of *A. fumigatus* including 1 strain of wild-type (WT), 1 strain of AF293, 27 strains of clinical *A. fumigatus* isolates (AF1 ~ AF27) and 2 strains of punctual mutation of the *Cyp51A* gene (TR34 and TR46), 13 strains of clinical *A. flavus* isolates (AFL1 ~ AFL13) and 1 strain of NRRL 3357, 4 strains of clinical *A. niger* isolates (AN1 ~ AN4), 5 strains of clinical *A. terreus* isolates (AT1 ~ AT5)] and 23 strains dematiaceous fungi [20 strains of *E. dermatitidis* (BMU00028-00041, 109140, 109145, 109149, D9g, D9h, D9i, D9j, D9k); 3 strains of *E. alcalophila* (CBS00017, CBS00038, CBSD0001)]. All strains were activated on Sabouraud Dextrose Agar (SDA) (Haibo Bio) for 2 to 3 days (37°C). All fungal strains were characterized through both microscopic examination of their morphological features and molecular identification via sequencing of the internal transcribed spacer (ITS) region of ribosomal DNA (rDNA) (Glass and Donaldson, 1995). For the precise identification of *Aspergillus* species, additional molecular analyses involving the sequencing of *β*-tubulin and calmodulin genes were performed (Hong et al., 2005; Samson and Varga, 2009). The *A. flavus* ATCC 204304 strain was used as a quality control strain in microdilution assays to ensure the accuracy of the minimum inhibitory concentration (MIC) determinations (Gao et al., 2024).

### Antifungal drugs and chemicals

BE (Catalog No. H2308245, Purity 98%), ITC (Catalog No. J2227367, Purity ≥98%), VRC (Catalog No. H2307623, Purity ≥98%), POS (Catalog No. H2224157, Purity ≥99%) and ISV (Catalog No. I337027, Purity ≥98%) five drugs were purchased from Aladdin Reagent Company in Shanghai, dissolved in dimethyl sulfoxide (DMSO, Macklin) to prepare the stock solution, resulting in a concentration of 3,200 μg/mL for BE and 6,400 μg/mL for azoles.

### Microdilution chequerboard technique

The antifungal drug solution was prepared according to the M38-A2 method issued by the Clinical Laboratory Standard Institute (CLSI) and previously published protocols (Gao et al., 2024). First, the activated filamentous fungi spores were suspended in PBS (Yisheng Bio), and the concentration was adjusted to 2 ~ 5 × 10^6^ spores/mL. The suspension was subsequently diluted to a concentration of approximately 1 ~ 3 × 10^4^ spores/mL for the filamentous fungi in RPMI 1640 liquid medium. Then, BE and azoles were diluted in RPMI 1640 liquid medium, with the final working concentration range being 0.5 ~ 32.0 μg/mL (for BE), 0.0625 ~ 8 μg/mL (for ITC and VRC) and 0.03125 ~ 4 μg/mL (for POS and ISV). In each direction of the 96-well plate, 50 μL of the diluted drug was added to form different concentration combinations of drugs, followed by the inoculation of the adjusted spore suspension into the 96-well plate, with 100 μL per well. Interpretation of results was performed after incubation at 35°C for 48 h for *Aspergillus*, and for 72 h for dematiaceous fungi, in accordance with previously published relevant literature (Gao et al., 2024). The MIC was determined by observing the growth of colonies, with the MIC defined as the lowest concentration at which no fungal growth was observed by the naked eye. To assess the combined effect of BE and azoles, the fractional inhibitory concentration index (FICI) was calculated. The formula for FICI is: FICI = (Ac/Aa) + (Bc/Bb), where Ac and Bc are the MICs when used in combination, and Aa and Bb are the MICs when used alone. Based on the FICI value, the type of drug interaction can be determined: FICI≤0.5 indicates a synergistic effect, 0.5 < FICI≤4 indicates no interaction, and FICI>4 indicates an antagonistic effect. All experiments were repeated three times.

## Results

### *In vitro* interactions between BE and azoles against *Aspergillus*

The MIC for the individual agents tested were as follows: for BE, all values exceeded 32 μg/mL; for POS, the range was between 0.25 and 1 μg/mL; for ITC, the range spanned from 0.5 to 8 μg/mL; for VRC, the concentrations varied from 0.125 to 4 μg/mL; and for ISV, the MICs were between 0.25 and 4 μg/mL (Table 1). When BE was combined with azoles, the MIC ranges for the drug pairs with synergistic effects were reduced to: BE at 4 μg/mL, POS at 0.03125 μg/mL, and ITC at 0.125 μg/mL; no significant synergistic effects were observed for VRC and ISV. In a cohort of 54 *Aspergillus* strains, the synergistic effects of the combination of BE with azoles were observed in 47 strains (87.04%) for BE/POS and in 18 strains (33.33%) for BE/ITC, the FICI values were found to span the ranges of 0.25 to 0.5 and 0.3125 to 0.5, respectively. Conversely, no significant synergistic effects were noted for the combinations involving BE with VRC and BE with ISV.

**Table 1:** *In vitro* interactions between BE and azoles against *Aspergillus.*

Strains	MIC^a^ of drug (μg/mL)					MIC [A/B(μg/mL)] (FICI^b^)			
	Alone					In combination			
	BE	POS	ITC	VRC	ISV	BE/POS	BE/ITC	BE/VRC	BE/ISV
*A. fumigatus*									
AF293	>32	0.5	1	0.5	0.5	8/0.125(S)	16/0.25(S)	16/0.25(I)	32/0.25(I)
WT	>32	0.25	0.5	0.25	0.5	4/0.0625(S)	2/0.25(I)	1/0.125(I)	0.5/0.5(I)
AF1	>32	0.5	0.5	0.25	0.5	8/0.0625(S)	4/0.25(I)	0.5/0.25(I)	32/0.25(I)
AF2	>32	0.5	0.5	0.25	1	4/0.125(S)	4/0.25(I)	0.5/0.25(I)	8/0.5(I)
AF3	>32	0.25	0.5	0.5	1	16/0.0625(S)	0.5/0.5(I)	4/0.25(I)	0.5/1(I)
AF4	>32	0.25	0.5	0.5	1	16/0.0625(S)	0.5/0.5(I)	8/0.25(I)	4/0.5(I)
AF5	>32	0.5	0.5	0.25	1	4/0.125(S)	1/1(I)	0.5/0.25(I)	16/0.5(I)
AF6	>32	0.25	0.5	0.25	1	16/0.0625(S)	0.5/0.25(I)	0.5/0.25(I)	32/0.5(I)
AF7	>32	1	>8	2	4	8/0.5(I)	>32/>8(I)	0.5/2(I)	0.5/4(I)
AF8	>32	0.5	0.5	0.25	0.5	8/0.0625(S)	8/0.25(I)	0.5/0.25(I)	0.5/0.5(I)
AF9	>32	0.25	1	0.5	0.5	8/0.0625(S)	16/0.25(S)	16/0.25(I)	0.5/0.5(I)
AF10	>32	0.25	0.5	0.25	0.5	8/0.0625(S)	16/0.125(S)	0.5/0.25(I)	16/0.25(I)
AF11	>32	0.5	0.5	0.25	0.5	8/0.125(S)	16/0.125(S)	1/0.125(I)	0.5/0.5(I)
AF12	>32	0.5	0.5	0.25	0.5	8/0.0625(S)	16/0.125(S)	0.5/0.25(I)	4/0.25(I)
AF13	>32	0.5	0.5	4	0.5	8/0.125(S)	16/0.25(I)	0.5/4(I)	32/0.25(I)
AF14	>32	0.25	0.5	0.5	0.5	8/0.125(I)	16/0.25(I)	16/0.25(I)	0.5/0.5(I)
AF15	>32	0.5	0.5	0.5	1	8/0.125(S)	8/0.25(I)	0.5/0.5(I)	32/0.5(I)
AF16	>32	0.25	1	0.25	0.5	8/0.0625(S)	16/0.25(S)	0.5/0.25(I)	4/0.25(I)
AF17	>32	0.5	0.5	0.25	0.5	4/0.125(S)	8/0.25(I)	0.5/0.25(I)	8/0.25(I)
AF18	>32	0.25	0.5	0.125	0.25	4/0.0625(S)	16/0.125(S)	32/0.0625(I)	32/0.125(I)
AF19	>32	0.25	1	0.25	0.5	8/0.0625(S)	16/0.25(S)	0.5/0.25(I)	32/0.25(I)
AF20	>32	0.25	0.5	0.125	0.25	8/0.03125(S)	8/0.125(S)	0.5/0.125(I)	32/0.125(I)
AF21	>32	0.25	0.5	0.25	0.25	4/0.0625(S)	4/0.25(I)	8/0.125(I)	0.5/0.5(I)
AF22	>32	0.25	1	0.25	0.5	4/0.0625(S)	16/0.25(S)	32/0.125(I)	32/0.125(I)
AF23	>32	0.25	1	0.25	0.5	8/0.125(I)	16/0.25(S)	0.5/0.5(I)	0.5/0.5(I)
AF24	>32	0.25	0.5	0.25	0.5	8/0.0625(S)	0.5/0.5(I)	0.5/0.25(I)	16/0.25(I)
AF25	>32	0.5	1	0.25	0.5	8/0.0625(S)	2/0.5(I)	0.5/0.25(I)	32/0.25(I)
AF26	>32	0.25	1	0.25	0.5	8/0.0625(S)	16/0.5(I)	0.5/0.25(I)	16/0.25(I)
AF27	>32	0.25	1	0.25	0.5	8/0.0625(S)	4/0.5(I)	0.5/0.25(I)	8/0.25(I)
TR46	>32	1	>8	4	>8	0.5/1(I)	>32/>8(I)	0.5/4(I)	>32/>8(I)
TR34	>32	1	>8	1	>8	0.5/1(I)	>32/>8(I)	0.5/1(I)	>32/>8(I)
*A. flavus*									
AFL1	>32	0.5	1	0.5	0.5	8/0.125(S)	8/0.5(I)	0.5/0.5(I)	0.5/0.5(I)
AFL2	>32	0.5	0.5	0.5	0.5	8/0.125(S)	0.5/0.5(I)	0.5/0.5(I)	0.5/0.5(I)
AFL3	>32	0.5	1	0.5	1	8/0.125(S)	2/0.5(I)	1/1(I)	0.5/1(I)
AFL4	>32	0.5	1	0.25	0.5	8/0.125(S)	8/0.5(I)	0.5/0.25(I)	0.5/0.5(I)
AFL5	>32	1	0.5	0.5	0.5	16/0.5(I)	4/0.5(I)	8/0.25(I)	1/1(I)
AFL6	>32	0.5	0.5	0.5	1	16/0.125(S)	2/0.25(I)	0.5/0.5(I)	0.5/1(I)
AFL7	>32	0.5	0.5	0.25	1	8/0.125(S)	8/0.125(S)	1/0.5(I)	0.5/1(I)
AFL8	>32	0.5	0.5	1	0.5	8/0.125(S)	4/0.25(I)	4/0.5(I)	0.5/0.5(I)
AFL9	>32	0.5	0.5	1	0.5	8/0.125(S)	8/0.125(S)	4/0.5(I)	0.5/0.5(I)
AFL10	>32	1	2	1	4	16/0.5(I)	4/4(I)	8/2(I)	0.5/4(I)
AFL11	>32	0.25	0.5	0.5	0.5	16/0.0625(S)	8/0.25(I)	0.5/0.5(I)	0.5/0.5(I)
AFL12	>32	0.25	0.5	0.5	0.5	8/0.0625(S)	16/0.125(S)	0.5/0.5(I)	0.5/0.5(I)
AFL13	>32	0.25	0.5	0.5	0.5	8/0.0625(S)	8/0.25(I)	0.5/0.5(I)	0.5/0.5(I)
NRRL 3357	>32	0.25	1	0.25	0.125	4/0.0625(S)	16/0.25(S)	16/0.125(I)	0.5/0.125(I)
*A. niger*									
AN1	>32	1	0.5	0.5	2	16/0.125(S)	4/1(I)	2/1(I)	0.5/2(I)
AN2	>32	0.5	0.5	0.25	1	16/0.125(S)	0.5/0.5(I)	32/0.5(I)	0.5/1(I)
AN3	>32	0.5	0.5	0.5	1	16/0.125(S)	8/2(A)	0.5/0.5(I)	4/2(I)
AN4	>32	1	0.5	0.5	2	16/0.25(S)	0.5/0.5(I)	16/1(I)	0.5/2(I)
*A. terreus*									
AT1	>32	0.5	0.5	0.5	1	8/0.0625(S)	8/0.125(S)	2/0.25(I)	2/0.5(I)
AT2	>32	0.5	0.25	0.125	0.5	8/0.0625(S)	4/0.125(I)	0.5/0.125(I)	0.5/0.5(I)
AT3	>32	0.5	0.5	0.25	0.5	8/0.0625(S)	8/0.125(S)	0.5/0.25(I)	8/0.25(I)
AT4	>32	0.5	0.5	0.25	0.5	8/0.0625(S)	4/0.125(S)	16/0.125(I)	8/0.25(I)
AT5	>32	0.25	0.5	0.25	0.25	8/0.03125(S)	0.5/0.5(I)	0.5/0.25(I)	8/0.125(I)
Quality control									
ATCC204304	>32	0.5	0.5	0.5	1	8/0.125(S)	2/0.25(I)	32/1(I)	0.5/1(I)

a The MIC is the concentration that inhibits 100% of growth.

b The FICI results are shown in parentheses.

S, synergy (FICI of ≤ 0.5); I, no interaction (indifference); (0.5 < FICI ≤ 4); A, antagonism (FICI of > 4).

### *In vitro* interactions between BE and azoles against dematiaceous fungi

*In vitro*, when tested against dematiaceous fungi, the MIC of BE was greater than 32 μg/mL. For POS, ITC, VRC and ISV, the MIC ranges were 0.125–1 μg/mL, 0.25–1 μg/mL, 0.0625–0.5 μg/mL, and 0.125–2 μg/mL, respectively (Table 2). When BE was combined with azoles, the MIC ranges for the drug pairs with synergistic effects were reduced to: BE at 4 μg/mL, POS at 0.03125 μg/mL, and ITC at 0.0625 μg/mL; no significant synergistic effects were observed for VRC and ISV. In a cohort of 23 dematiaceous fungi, synergistic effects of the combination of BE with azoles were observed in 22 strains (95.65%) for BE/POS and in 2 strains (8.70%) for BE/ITC, with FICI values ranging from 0.25 to 0.5 and 0.375, respectively. In contrast, antagonistic effects were noted in 6 strains (27.27%) for BE/ITC, 4 strains (18.18%) for BE/VRC, and 4 strains (18.18%) for BE/ISV, while the remainder exhibited no significant interaction.

**Table 2:** *In vitro* interactions between BE and azoles against dematiaceous fungi.

Strains	MIC^a^ of drug(μg/mL)					MIC [A/B(μg/mL)] (FICI^b^)			
	Alone					In combination			
	BE	POS	ITC	VRC	ISV	BE/POS	BE/ITC	BE/VRC	BE/ISV
*E. dermatitidis*									
BMU00028	>32	0.5	0.5	0.0625	0.25	8/0.125(S)	16/2(A)	32/0.25(A)	8/1(A)
BMU00029	>32	0.5	1	0.25	0.5	8/0.0625(S)	8/0.5(I)	32/0.5(I)	0.5/1(I)
BMU00030	>32	0.25	0.5	0.25	0.5	16/0.0625(S)	16/1(I)	32/0.5(I)	0.5/1(I)
BMU00031	>32	1	0.5	0.5	2	8/0.125(S)	16/0.25(I)	0.5/0.5(I)	32/1(I)
BMU00034	>32	0.25	1	0.125	2	8/0.0625(S)	0.5/0.5(I)	0.5/0.25(I)	0.5/2(I)
BMU00035	>32	0.25	0.5	0.125	0.5	8/0.0625(S)	8/0.25(I)	0.5/0.25(I)	0.5/1(I)
BMU00036	>32	0.25	0.5	0.0625	0.25	16/0.0625(S)	8/0.25(I)	0.5/0.125(I)	32/1(A)
BMU00037	>32	0.5	0.5	0.125	1	16/0.0625(S)	16/2(A)	0.5/0.25(I)	16/2(I)
BMU00038	>32	0.5	0.5	0.25	0.5	16/0.25(I)	16/1(I)	0.5/0.25(I)	0.5/1(I)
BMU00039	>32	0.5	0.5	0.125	0.5	8/0.0625(S)	32/1(I)	0.5/0.125(I)	0.5/1(I)
BMU00040	>32	0.125	0.25	0.0625	0.25	16/0.03125(S)	0.5/0.25(I)	0.5/0.0625(I)	0.5/0.5(I)
BMU00041	>32	0.25	0.5	0.0625	0.5	8/0.0625(S)	8/0.125(S)	8/0.25(A)	0.5/1(I)
109140	>32	0.25	0.5	0.25	2	16/0.0625(S)	16/2(A)	0.5/0.25(I)	0.5/2(I)
109145	>32	0.5	1	0.25	1	8/0.125(S)	0.5/0.5(I)	0.5/0.25(I)	0.5/1(I)
109149	>32	0.5	1	0.25	0.25	8/0.125(S)	16/8(A)	32/0.5(I)	1/1(A)
D9g	>32	0.5	0.5	0.25	0.5	16/0.0625(S)	16/4(A)	32/1(A)	8/2(A)
D9h	>32	0.5	0.5	0.125	1	16/0.0625(S)	4/0.25(I)	32/0.0625(I)	32/0.5(I)
D9i	>32	0.5	0.5	0.25	0.5	16/0.0625(S)	16/4(A)	16/1(A)	8/2(A)
D9j	>32	0.5	0.25	0.0625	0.25	8/0.0625(S)	2/0.125(I)	32/0.125(I)	0.5/0.25(I)
D9k	>32	0.5	0.5	0.25	0.5	8/0.125(S)	0.5/0.5(I)	0.5/0.25(I)	0.5/1(I)
*E. alcalophila*									
CBS00017	>32	0.125	0.25	0.0625	0.125	4/0.03125(S)	8/0.0625(S)	0.5/0.0625(I)	8/0.0625(I)
CBS00038	>32	0.25	0.25	0.25	0.5	16/0.0625(S)	8/0.5(I)	0.5/0.25(I)	0.5/0.5(I)
CBSD0001	>32	0.125	0.25	0.0625	0.25	4/0.03125(S)	4/0.125(I)	0.5/0.0625(I)	0.5/0.5(I)

a The MIC is the concentration that inhibits 100% of growth.

b The FICI results are shown in parentheses.

S, synergy (FICI of ≤ 0.5); I, no interaction (indifference); (0.5 < FICI ≤ 4); A, antagonism (FICI of > 4).

### Summary of *in vitro* interactions between BE and azole against fungi

**Figure 1:**
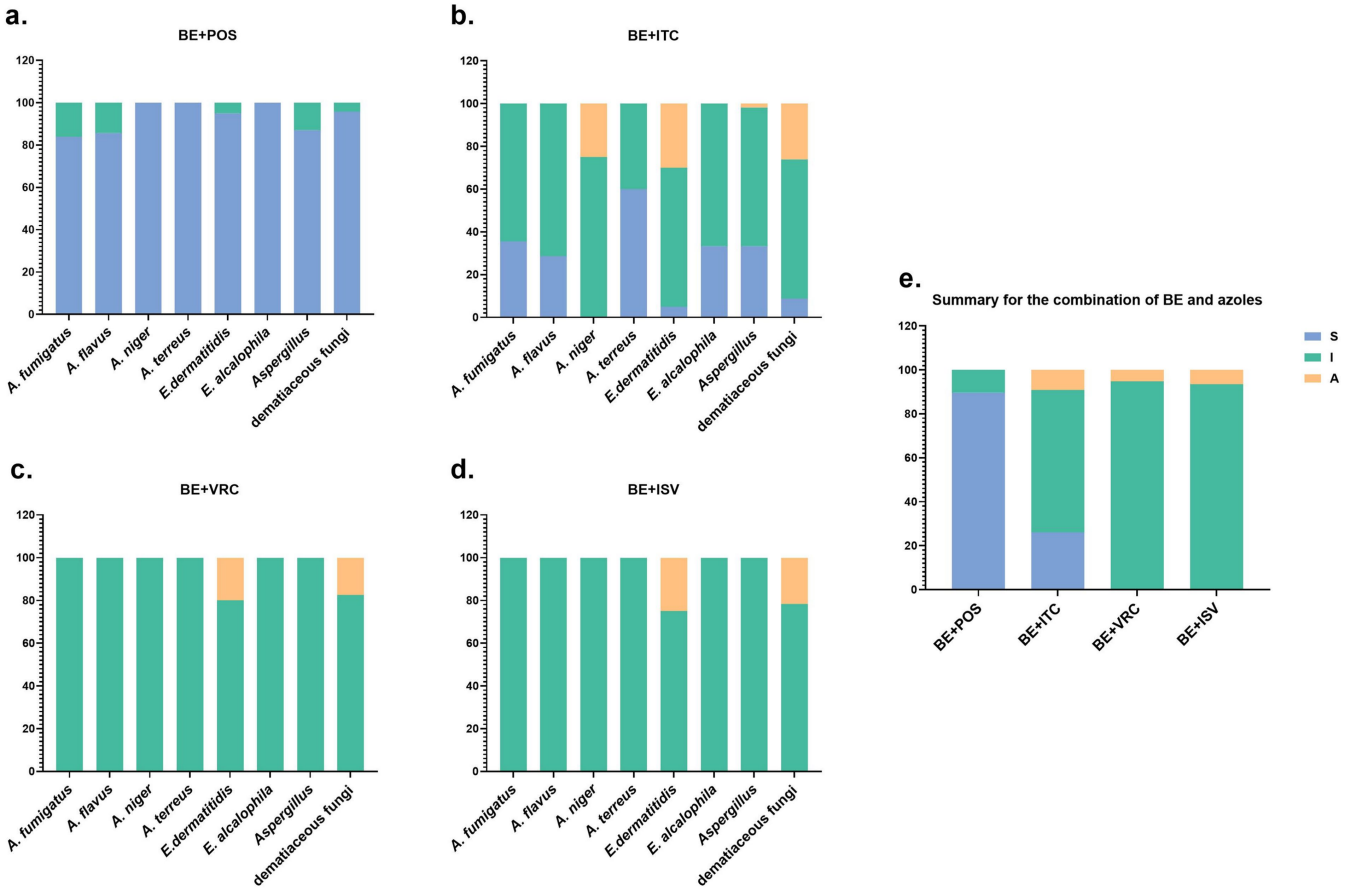
Summary of drug interaction for the combination of BE and azoles. **(a–d)** The fraction of in vitro interaction results of BE combined with POS, ITC, VRC, and ISV antifungal agents, respectively. **(e)** Summary of interaction relationships for all drug combinations against all fungi. S, synergy (FICI of ≤ 0.5); I, no interaction (indifference)(0.5 < FICI ≤ 4); A, antagonism (FICI of > 4).

The *in vitro* interaction study of BE in combination with POS antifungal agents revealed synergistic effects against *Aspergillus* species, with 26 out of 31 *A. fumigatus* strains (83.87%), 12 out of 14 *A. flavus* strains (85.71%), all 4 *A. niger* strains, and 5 out of 5 *A. terreus* strains exhibiting such effects (Figure 1a). Among the dematiaceous fungi, 19 out of 20 *E. dermatitidis* strains (95%) and all 3 *E. alcalophila* strains demonstrated synergistic activity. 7 *Aspergillus* strains and one dematiaceous fungi strain exhibited no interaction.

In the *in vitro* interaction study of BE combined with ITC antifungal agents, synergistic effects were observed in *Aspergillus* species, with 11 out of 31 *A. fumigatus* strains (35.48%), 4 out of 14 *A. flavus* strains (28.57%), and 3 out of 5 *A. terreus* strains (60%) exhibiting such effects (Figure 1b). Among the dematiaceous fungi, 1 out of 20 *E. dermatitidis* strains (5%) and 1 out of 3 *E. alcalophila* strains (33.33%) demonstrated synergistic activity. 35 *Aspergillus* strains and 15 dematiaceous fungi strains showed no interaction, 1 *Aspergillus* strain and 6 dematiaceous fungi strains displayed antagonistic effects.

In the combinations of BE with VRC and ISV, all 54 *Aspergillus* strains exhibited no interaction (Figures 1c,d). Among the dematiaceous fungi, there were 19 strains with no interaction with BE and VRC, and 18 strains with no interaction with BE and ISV. Additionally, 4 strains showed an antagonistic effect with the BE and VRC combination, and 5 strains with the BE and ISV combination.

In the panel of 77 tested fungal strains, 69 (89.61%) demonstrated synergistic interactions in response to the drug combination of BE and POS, while 20 (25.97%) showed synergistic effects with the BE and ITC combination (Figure 1e). No synergistic interactions were observed with BE in combination with VRC or ISV.

## Discussion

Results indicate that among the azoles, combinations of BE with POS and ITC demonstrated synergistic effects against the tested fungal strains. Notably, the BE/POS combination exhibited the most pronounced synergistic effect, observed in 89.61% of strains, with a more frequent observation of synergy in dematiaceous fungi compared to *Aspergillus*. In contrast, the BE/ITC combination showed significantly less synergy, affecting only 25.97% of strains. The disparity in synergistic effects between the BE/POS and BE/ITC combinations may be attributed to differences in the chemical structures and mechanisms of action of these azoles.

At a concentration of 0.25 mM, BE alleviates *A. fumigatus* keratitis in mice by inhibiting fungal growth, biofilm formation and adhesion, and by downregulating the expression of pro-inflammatory factors (Zhu et al., 2021). For *C. albicans*, BE inhibits fungal growth by targeting *Eno1*, inhibiting glycolysis, and preventing biofilm formation (Li L. et al., 2022). Treatment with BE also induces concentration-dependent accumulation of ROS in *Trichophyton mentagrophytes* and *C. albicans*. When BE is used in combination with FLU, it demonstrates robust antifungal activity against drug-resistant fungi. In this context, the biofilm formation of *C. albicans* is inhibited in a dose-dependent manner at concentrations ranging from 4 to 32 μg/mL (Huang et al., 2008). Baicalein-Core Derivatives can also enhance the antifungal efficacy of FLU by inhibiting hyphal formation in *C. albicans* (Zhou et al., 2023). The antifungal mechanisms of BE may involve inhibiting biofilm formation and inducing the accumulation of ROS. Further research is needed to elucidate the specific antifungal mechanisms at the molecular level. Azoles inhibit fungal growth by blocking ergosterol synthesis through the inhibition of 14α-sterol demethylase (*CYP51*) in the fungal cell membrane, leading to impaired cell membrane biogenesis and altered membrane permeability (Patterson et al., 2016). In this study, the combination of BE with POS and ITC exhibited synergistic effects against the tested fungi. In contrast, no synergy was observed when BE was combined with VRC and ISV. Previous studies using molecular docking and molecular dynamics simulations to investigate the binding mechanisms and tunneling characteristics of *CYP51* with inhibitors have shown that hydrophobic interactions are the primary driving force for binding to *CYP51*, and that long-chain inhibitors such as POS and ITC can access more *CYP51* residues through hydrophobic interactions than short-chain inhibitors like VRC, thereby exhibiting stronger binding affinities (Shi et al., 2020). ISV is a novel azole drug that is structurally similar to VRC (Ghobadi et al., 2022). These differences in binding affinity may account for the lack of synergy observed with ISV and VRC.

Current research on the toxicity of BE is relatively limited. At doses cytotoxic to malignant cells, BE displays minimal or negligible toxicity to normal peripheral blood cells and normal myeloid cells, but it also exerts growth-inhibitory effects on human fetal lung diploid cell lines at the same concentrations that suppress tumor cell proliferation (Li-Weber, 2009). Preliminary animal studies have indicated that BE exhibits low acute toxicity at therapeutic doses, with no significant adverse reactions observed (Wang et al., 2022). In clinical studies involving healthy Chinese subjects, both single-dose and multiple-dose administrations of BE tablets have demonstrated good safety and tolerability, with no serious or severe adverse reactions reported (Li et al., 2021). Studies have shown that the combination of BE at a concentration of 32 μg/mL with ampicillin has negligible effects on hemolysis of red blood cells (RBCs) and cytotoxicity towards Vero cells. This concentration falls within the range tested in our MIC assays (Lu et al., 2021). However, a more comprehensive toxicological evaluation is warranted, especially in the context of the interactions between BE and azoles, to establish the safety profile of these drug combinations through *in vitro* cytotoxicity assays and *in vivo* animal models.

The study also noted antagonistic effects in certain cases, particularly with combinations of BE/ITC, BE/VRC, and BE/ISV. These antagonistic effects may arise from competitive inhibition or other unknown molecular interactions that negate the antifungal activity. Additional research is required to understand and potentially mitigate these antagonistic effects.

## Data Availability

The original contributions presented in the study are included in the article/supplementary material, further inquiries can be directed to the corresponding authors.
